# Schlafen2 mutation unravels a role for chronic ER stress in the loss of T cell quiescence

**DOI:** 10.18632/oncotarget.9818

**Published:** 2016-06-03

**Authors:** Ibrahim Omar, Antonio Lapenna, Leonor Cohen-Daniel, Boaz Tirosh, Michael Berger

**Affiliations:** ^1^ The Lautenberg Center for Immunology and Cancer Research, The Biomedical Research Institute Israel-Canada of the Faculty of Medicine, The Hebrew University Hadassah Medical School, Jerusalem, Israel; ^2^ Institute for Drug Research, School of Pharmacy, Faculty of Medicine, Hebrew University of Jerusalem, Jerusalem, Israel

**Keywords:** ER stress, UPR, Slfn2, quiescence, XBP1, Immunology and Microbiology Section, Immune response, Immunity

## Abstract

Immunologically naïve lymphocytes are kept in a quiescent state until antigen engagement. These quiescent immune cells are characterized by small cell size, lack of spontaneous proliferation and low metabolic rate. Lymphocyte quiescence is actively enforced condition which ensures the preservation of proper differentiation and proliferation capabilities of naïve and memory lymphocytes. Previously we described a chemically induced mutation in Schlafen2 (Slfn2), termed *elektra*, which breaks quiescence and compromises immunity. However, the mechanism by which Slfn2 maintains quiescence remains unknown. Here we demonstrate that *elektra* T cells display chronic ER stress under steady state conditions. Modulation of ER stress response by depletion of either UPR mediators XBP1 or CHOP, improved viability and partially corrected the developmental abnormalities and proliferation capabilities of *elektra* T cells. Altogether, our results demonstrate a functional connection between Slfn2 induced quiescence in T cells and ER homeostasis, clarifying a novel mechanism by which immune cell quiescence is maintained.

## INTRODUCTION

The endoplasmic reticulum (ER) is the entry site of proteins into the secretory pathway and a central organelle for lipid synthesis. To acquire a native state in the ER, newly translated proteins undergo post translation modifications and are engaged by molecular chaperones. A disruption of ER homeostasis by physiological, pathogenic or chemical insults leads to the accumulation of unfolded or misfolded proteins, conditions termed ER stress [1]. ER stress elicits an unfolded protein response (UPR), a signaling pathway that emanates from the ER, aimed to restore ER function [2]. The UPR is mediated through the activation of three ER transmembrane stress sensors; pancreatic ER kinase (PKR)-like ER kinase (PERK), activating transcription factor 6 (ATF6) and inositol-requiring enzyme 1 (IRE1) [3]. These proteins are maintained in an inactive state through interaction with the ER chaperone, glucose regulated protein 78 (GRP78, also known as Bip). Unfolded proteins can interact directly with IRE1 to mediate its activation or trigger the release of GRP78 from the UPR sensors allowing their activation [4]. Activated IRE1, PERK and ATF6 promote adaptation to ER stress through the activation of transcriptional upregulation of key ER chaperones as well as by controlling protein synthesis through the phosphorylation of eIF2α. The transcriptional arm of the UPR adjusts ER folding capacity, enhances ER-associated degradation (ERAD) and drives the expansion of ER volume [5]. However, if ER stress is not alleviated in a timely manner, the UPR promotes apoptosis via multiple mechanisms. The switch from pro-survival to pro-apoptotic roles depends on the cell type and the type of insults [6]. One of the key factors in maintaining this balance is the activation of the transcription repressor CHOP, of which level is controlled by all UPR arms, primarily by PERK through ATF4 induction [7]. CHOP has been shown to alter the balance between pro-survival and pro-apoptotic Bcl-2 family members [8, 9] and death receptor 5 (DR5) thus promoting apoptosis through the mitochondrial pathway [10]. Depletion of CHOP in both animal models and cell lines showed a significant reduction in ER stress-induced apoptosis [7]. IRE1 possess both pro-survival and pro-death roles. Dependent on its kinase activity, IRE1 activates TNF receptor associated factor 2 (TRAF2) and JNK, which ultimately signals to apoptosis. This activity of IRE1 occurs in parallel to its nuclease activity which mediates the splicing of XBP1 mRNA to generate the transcriptionally active form of XBP1 protein. Depending on the stress strength and persistency as well as on the cell type, active XBP1 can drive either pro-survival or pro-death transcriptional networks [11–15]. XBP1 mRNA is not the only substrate for IRE1 nuclease activity. A growing list of mRNA and miRNA molecules was found to be targeted for degradation by IRE1, in a manner critical to cell survival [16]. Importantly, constitutive ER stress conditions, termed chronic ER stress, usually arise on the background of severe disturbance of homeostasis and are rarely seen physiologically.

Survival of naive newly thymic emigrants T cells is dependent on their ability to maintain quiescence. Quiescent immune cells are characterized by small cell size, lack of spontaneous proliferation and low metabolic rate [17–19]. Acquisition of this state is important to provide stress free environment and resistance to apoptosis while keeping the naïve T cells responsive to activating stimuli [17, 19–22].

Previously, we described a mouse strain bearing a Slfn2-mutated allele, named *elektra*. The *elektra* mutation causes an isoleucine-to-asparagine substitution of residue 135 of the 278 amino acid of SLFN2 protein [23]. Using these mice we demonstrated an essential regulatory role for SLFN2 in both innate and adaptive immune responses [23].

In *elektra* mutant mouse, naïve newly thymic emigrant (CD44^lo^) fail to maintain quiescence and instead acquire a semiactivated phenotype characterized by activation of part of JNK and p38, higher propensity to enter cell cycle as well as downregulation of IL7Ra and CD62L [23]. As a result, upon maturation (CD44^hi^) or activation signals, *elektra* T cells fail to acquire memory-like phenotype and to engage pro-survival machinery leading to premature apoptosis. In addition to T cells, inflammatory monocytes are also affected by the *elektra* mutation, exhibiting similar fragility in the face of signals of proliferation or activation [23]. A recent study from our group showed an essential role for Slfn2 in the progression of T cell malignancies such as T-ALL and lymphoma as well as in other diseases evolving aberrant T cell development [24]. These findings highlight the great potential in targeting Slfn2 and other family members for therapeutic purposes, either to manipulate specific immune responses or to suppress blood borne malignancies. However, the mechanism by which Slfn2 maintains quiescent, stress-free environment in T cells is still unknown.

In the present study, we demonstrate that *elektra* monocytes and T cells exhibit chronic ER stress conditions. By partially preventing the engagement of the UPR response either by CHOP or XBP1 depletion, viability of *elektra* cells was restored and proliferation capabilities of *elektra* T cells were improved. These results establish for the first time a functional connection between the loss of quiescence in Slfn2-deficiency to chronic unresolved ER stress.

## RESULTS

### ER stress regulated genes are elevated in *elektra* monocytes

In *elektra* cells both JNK and the p38 pathways are constitutively active without the activation of the ERK1/2 pathway. This phenotype is typical to a variety of stress conditions, such as starvation, ER stress, DNA damage and oxidative stress [23].

**Figure 1:**
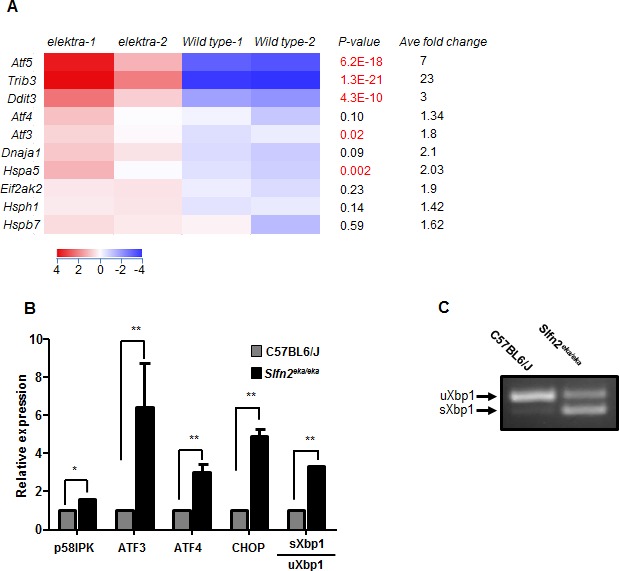
High expression of ER stress related genes in *elektra* inflammatory monocytes **A**. Heat map of cDNA expression array shows differentially expressed genes in C57BL6/J and Slfn2^*eka/eka*^ bone marrow CD11b^*+*^ ly6C^*hi*^ sorted monocytes. *P-values* (highly significant are marked by red font) and average fold of change are included. **B**. Gene expression analyzed by semi-quantitative RT-PCR of ER stress response genes in C57BL6/J and Slfn2^*eka/eka*^ bone marrow CD11b^*+*^ ly6c^*hi*^ monocytes (*n=4*). **C**. Representative result of XBP1 splicing in Slfn2^*eka/eka*^ monocytes. Total RNA extracted from sorted monocytes and subjected to RT-PCR analysis with XBP1 primers.

To identify which of the stress conditions is responsible for the aberrant activation of the MAPK pathway in *elektra* cells, we performed an unbiased transcriptome profiling. To avoid possible secondary defects, such as activation of apoptotic signaling pathway mediated by the *elektra* mutation, we decided to analyze monocyte precursors (CD11b^+^/ly6C^hi^) from the bone marrow. These cells are phenotypically normal and viable in *elektra* mice [23]. The gene expression profile of the BM monocytes precursors clearly shows elevated levels of cell stress related genes, particularly ER stress, in *elektra* cells as compare to cells from wild-type mice. Among these are genes coding for members of activating transcription factors/cAMP response element binding protein (ATF/CREB) family; ATF3, ATF4 and ATF5 [25, 26]. Up-regulation of these genes has been strongly related to cellular stresses, survival and cell death. Additionally, components of ER stress mediated apoptosis pathway i.e. C/EBP homologous protein (CHOP/DDIT3/GADD153)[8] and TRIB3 [27], an Akt inhibitor, were found to be significantly enriched in *elektra* cells. Interestingly, during ER stress, CHOP and TRIB3 are induced by ATF4 [27] which is also induced in *elektra* cells as mentioned above. Furthermore, we also observed up regulation of several chaperones; Hspa5 (encodes for the ER chaperone Bip), Hspb7, Hsph1 and the co-chaperone Dnaja1 (Hsp40) in *elektra* cells, emphasizing up regulation of the UPR and ER stress [28]. Finally, our results show elevated level of the protein synthesis regulator, Eif2ak2, which phosphorylates and inhibits the translation initiation factor eIF2α leading to translation inhibition, an essential process in UPR [29]. Microarray results were validated by real time PCR (Figure 1B).

The splicing of XBP1 mRNA is a hallmark of ER stress. Semi quantitative real time-PCR analysis for XBP1 splicing (Figure 1B), which was also confirmed by PCR analysis for the spliced and unspliced forms of XBP1 (Figure 1C), demonstrated a constitutive level of the spliced form, condition that is rarely seen for unstimulated cells. These results demonstrate that the *elektra* mutation in Slfn2 leads to the unabated activation of stress response in BM monocytes.

### Chronic ER stress of *elektra* T cells

**Figure 2:**
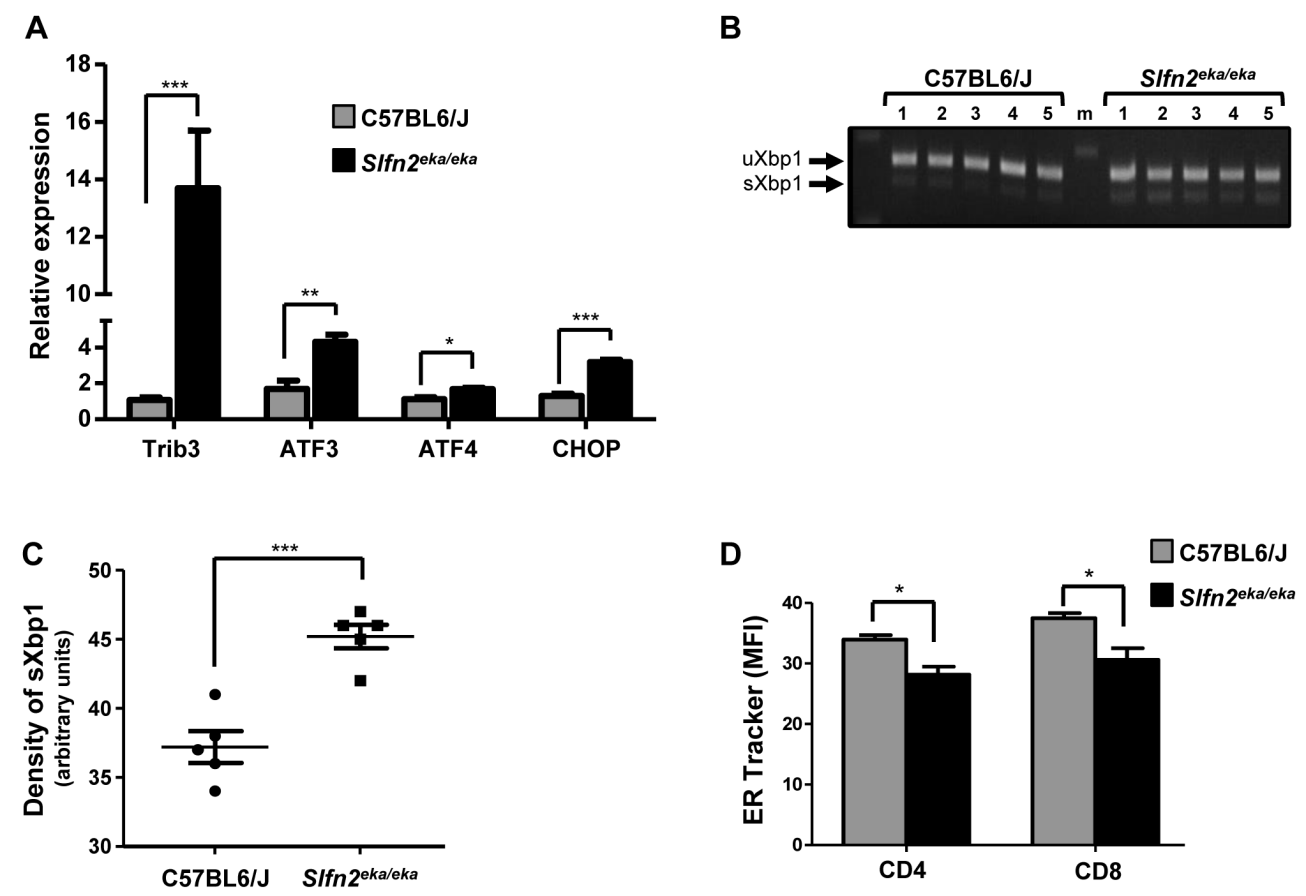
Activation of UPR and ER stress response in Slfn2^*eka/eka*^ T cells **A**. Gene expression of ER stress response and UPR genes in naïve T cells purified from C57BL6/J and Slfn2^*eka/eka*^ splenocytes (*n=4* each, 96-98% and 95-96% purity of wt and *elektra* T cells respectively). **B**. XBP1 splicing in C57BL6/J and *Slfn2*^*eka/eka*^ T cells (5 different mice from each genotype). **C**. Scatter plot presenting densitometry of the bands in B. **D**. ER tracker blue white dpx staining of both CD4^*+*^ and CD8^*+*^ T cells obtained from C57BL6/J or Slfn2^*eka/eka*^ mice (*n=3* each).

Next we aimed to confirm that the ER stress response is activated also in *elektra* T cells. For this purpose T cells were isolated from *elektra* spleens and the expression of ER stress genes was measured in comparison to wt cells. ER stress response genes were indeed elevated in *elektra* T cells (Figure 2A), and the ratio between the spliced and unspliced transcripts of XBP1 was elevated (Figure 2B). In addition, ER tracker staining shown to be decreased in *elektra* T cells as compare to wt T cells (Figure 2C) suggesting a defect in ER structure. These data confirm that similarly to *elektra* monocytes, ER stress response is activated also in *elektra* T cells.

### *Elektra* T cells die via ER stress induced apoptosis

*Elektra* mice overexpressing *BCL2* in the T cell compartment, *BCL2(Tg)/Slfn2^eka/eka^* have normal numbers of T cells. In addition, Bcl2 is downregulated in *elektra* T cells [23, 24]. These results demonstrate that *elektra* T cells undergo apoptosis via the intrinsic apoptotic pathway [23, 24]. Since chronically activation of ER stress promotes apoptosis in several cell types [30], we postulated a contribution of this pathway in the demise of *elektra* T cells.

**Figure 3:**
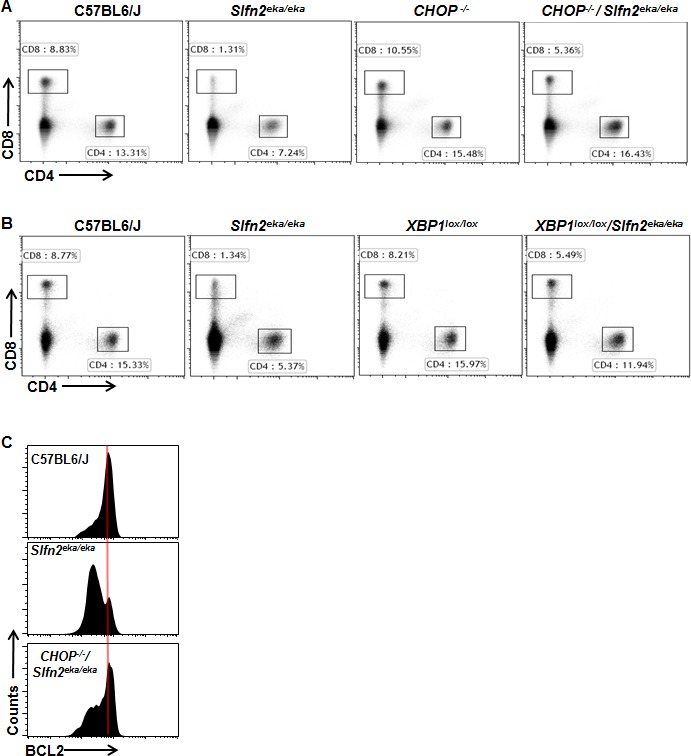
CHOP or XBP1 deficiency rescues the death of *Slfn2*^*eka/eka*^ CD4^**+**^ and CD8^+^ T cells **A**., **B**. Flow cytometry analysis of the expression of CD4 and CD8 by cells from the spleen of wild-type, *Slfn2*^*eka/eka*^, *CHOP*^*−/−*^ and *CHOP*^*−/−*^ */Slfn2*^*eka/eka*^ (A) or *XBP1*^*lox/lox*^ and *XBP1*^*lox/lox*^ */Slfn2*^*eka/eka*^ (B) (n=9). Numbers in gates indicate percent cells in each. **C**. Flow cytometry of BCL2 expression in splenic CD8^*+*^CD44^*hi*^ cells from C57BL6/J, *Slfn2*^*eka/eka*^ or *CHOP*^*−/−*^ */Slfn2*^*eka/eka*^ mice (n=4 each genotype).

To test this possibility, we examined whether *elektra* T cell death is indeed mediated by chronic ER stress by evaluating whether modulation of the ER stress response by either down regulation of XBP1 or CHOP can rescue the *elektra* T cell phenotype. To this end, *Lck-Cre XBP1^lox/lox^* mice, in which XBP1 is knocked out specifically in the T cell compartment, or CHOP knockout mice, *CHOP^−/−^*, were crossed to *elektra* mice to generate *Lck-Cre XBP1^lox/lox^/Slfn2^eka/eka^* or *CHOP^−/−^/Slfn2^eka/eka^* transgenic mice respectively. The percentages of CD4^+^ and CD8^+^ T cells from spleens of *Lck-Cre XBP1^lox/lox^/Slfn2^eka/eka^* or *CHOP^−/−^/Slfn2^eka/eka^* transgenic mice were markedly elevated as compare to those from *elektra* mice, in fact resembling those of wild-type mice (Figure 3A and 3B). In addition and in line with these results, BCL2 expression levels were restored in CD8^+^ T cells from *CHOP^−/−^/Slfn2^eka/eka^* mice (Figure 3C). Our data implicate the XBP1/CHOP pathway as mediator of *elektra* T cell death.

### Blocking ER stress response partially restores *elektra* T cell semiactivated phenotype

Viability is not the only defect of *elektra* T cells. Previously we showed that *elektra* T cells exist in a semiactivated state, characterized by down regulation of the IL7Ra (CD127) and the homing receptor L-Selectin (CD62L) on both naïve newly thymic emigrant (CD44^lo^) and on naïve mature (CD44^hi^) T cells. In addition, the mature CD44^hi^ *elektra* T cells fail to acquire a memory-like phenotype (CD122^+^) and instead develop into recently-activated (CD122^−^) T cells [23].

BCL2 overexpression [23] or p53 deficiency [24] rescue *elektra* T cell death, but does not affect their semiactivated phenotype, and thus does not correct their proliferation defect [23]. These results illustrate the complexity of the *elektra* phenotype and indicate that apoptosis of the *elektra* T cells is a consequence of their acquisition of a semiactivated state.

Therefore, we next assessed whether manipulating the ER stress response by CHOP or XBP1 deficiency also impinges on the acquisition of the semiactivated state of the *elektra* T cells and their proliferation capabilities. To this end T cells from *Lck-Cre XBP1^lox/lox^/Slfn2^eka/eka^* and *CHOP^−/−^/Slfn2^eka/eka^* mice were further immunophenotyped by flow cytometry.

**Figure 4:**
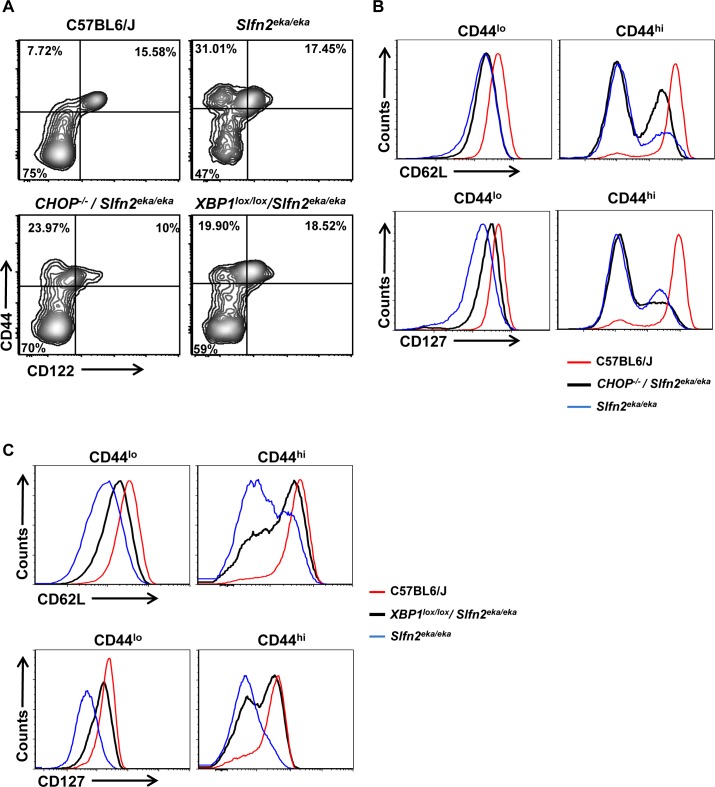
CHOP or XBP1 deficiency partially rescues the semiactivated phenotype of *Slfn2*^*eka/eka*^ CD8^**+**^ T cells **A**. Flow cytometry analysis of the staining for CD122 (IL-2Rβ) and CD44 in splenic CD8^*+*^ T cells from C57BL6/J, *Slfn2*^*eka/eka*^, *CHOP*^*−/−*^ */Slfn2*^*eka/eka*^ or *XBP1*^*lox/lox*^ */Slfn2*^*eka/eka*^ mice (n =9). Numbers in quadrants indicate percent cells in each. **B**., **C**. Flow cytometry analysis for the expression levels of CD62L (upper panel) and IL-7Rα (CD127) (lower panel) on surface of both CD8^*+*^CD44^*lo*^ (left) and CD8^*+*^CD44^*hi*^ (right) T cells from the C57BL6/J, *Slfn2*^*eka/eka*^, *CHOP*^*−/−*^ */Slfn2*^*eka/eka*^ (B) or *XBP1*^*lox/lox*^ */Slfn2*^*eka/eka*^ mice (C) (*n=9*).

Similar to *elektra* T cells, both *Lck-Cre XBP1^lox/lox^/Slfn2^eka/eka^* and *CHOP^−/−^/Slfn2^eka/eka^* CD44^hi^ CD8^+^ and CD4^+^ T cell populations failed to gain a memory-like phenotype of CD44^hi^/CD122^+^, and were mostly CD122^−^ or CD122^lo^ (Figure 4A and Supplementary Figure 1A). Comparable to *elektra,* in *CHOP^−/−^/Slfn2^eka/eka^* most of this CD44^hi^/CD122^−/lo^ population display a complete shedding of CD62L (L-selectin) (Figure 4B and Supplementary Figure 1B, right upper panel), and no surface expression of IL-7 receptor α-chain (IL-7Rα or CD127) (Figure 4B and Supplementary Figure 1B, right lower panel). In *Lck-Cre XBP1^lox/lox^/Slfn2^eka/eka^* mice most of the CD44^hi^CD122^lo/−^ T cell population showed partial restoration of both CD62L (Figure 4C and Supplementary Figure 1C, right upper panel) and CD127 (Figure 4C and Supplementary Figure 1C, right lower panel) expression. In addition, the CD44^lo^ (naive) population of CD8^+^ and CD4^+^ T cells from both, *CHOP^−/−^/Slfn2^eka/eka^* and *Lck-Cre XBP1^lox/lox^/Slfn2^eka/eka^*, mice partially restored the expression of both CD62L (Figure 4B and 4C and Supplementary Figure 1B and C, left upper panels) and CD127 (Figure 4B and 4C and Supplementary Figure 1B and C, left lower panels). These results suggest that the UPR through XBP1, and to lesser extent by CHOP, compromise peripheral T cell maturation and ability to maintain quiescence.

### Blocking ER stress response partially restores *elektra* T cell proliferation capacity

**Figure 5:**
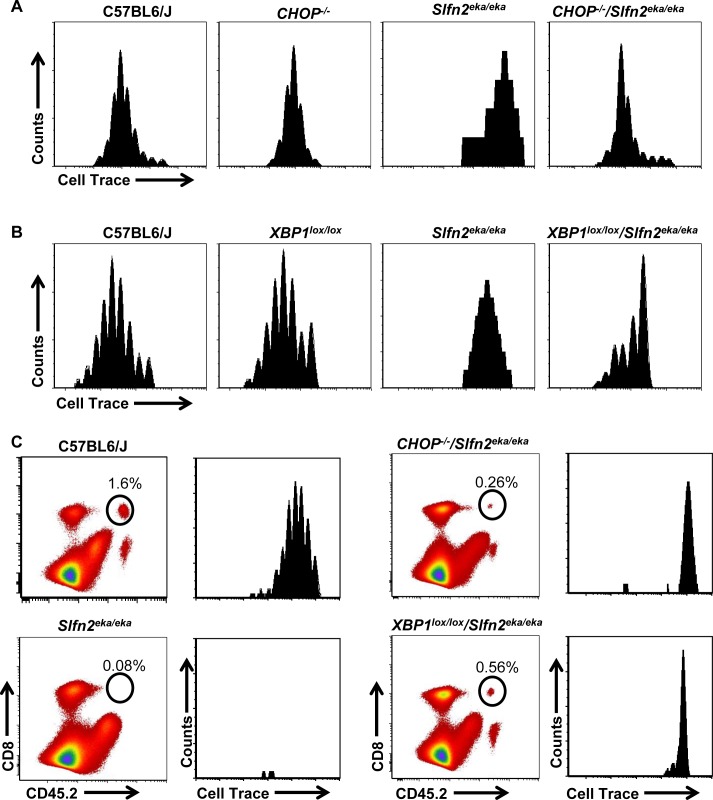
CHOP or XBP1 deficiency partially rescues *Slfn2*^*eka/eka*^ CD8^**+**^ T cells proliferation capacity and death in lymphopenic environment *in vivo* **A**., **B**. Cell trace dilution of splenic CD8 T cells obtained from C57BL6/J, *Slfn2*^*eka/eka*^, *CHOP*^*−/−*^ */Slfn2*^*eka/eka*^ (A) or *XBP1*^*lox/lox*^ */Slfn2*^*eka/eka*^ (B) mice stimulated for 72 hours with plate bounded anti-CD3ε (2μg/ml) plus anti-CD28 (1μg/ml) and IL-2 (20ng/ml) (*n=6* each genotype). Histograms are gated on CD8^+^. **C**. Dot plot of CD45.2 versus CD8 (left panels) and histograms presenting Cell trace dilution of splenic CD8^+^ (right panels) obtained from CD45.2 C57BL6/J, *Slfn2*^*eka/eka*^, *CHOP*^*−/−*^ */Slfn2*^*eka/eka*^ or *XBP1*^*lox/lox*^ */Slfn2*^*eka/eka*^ adoptively transferred into irradiated (400 rad) CD45.1 mice for 7 days (*n=5*). Numbers on plots indicate percentages of donor survived CD8^+^ T cells. Histograms are gated on CD45.2^+^ CD8^+^.

Next we examined the impact of ER stress on the proliferative capacity of *elektra* T cells. Initially, we examined the proliferation capacity of *CHOP^−/−^/Slfn2^eka/eka^* or *Lck-Cre XBP1^lox/lox^/Slfn2^eka/eka^* T cell in response to activation signals. For this end, wt, *CHOP^−/−^/Slfn2^eka/eka^*, *Lck-Cre XBP1^lox/lox^*/*Slfn2^eka/eka^* and *elektra* splenic T cells were labeled with the cytosolic dye Cell Trace and were stimulated for 72 h *in vitro* with a combination of antibodies to CD3ε and CD28. In line with our published data [23], *elektra* T cells completely failed to proliferate in response to activation stimuli (Figure 5A and 5B and, Supplementary Figure 2A and B). However, as opposed to *elektra*, both CD8^+^ and CD4^+^ T cells from *CHOP^−/−^/Slfn2^eka/eka^* and *Lck-Cre XBP1^lox/lox^*/*Slfn2^eka/eka^* had undergone proliferation, although less than T cells from *CHOP*^−/−^ and *Lck-Cre XBP1^lox/lox^* that proliferated comparably to the T cells from wt mice (Figure 5A and 5B and, Supplementary Figure 2A and B). These results demonstrate that the proliferation defect of *elektra* T cells upon activation ex vivo is mediated, at least in part, by the chronic ER stress.

To further test the impact of the elevated ER stress on the proliferative capacity of *elektra* T cells, we examined *CHOP^−/−^/Slfn2^eka/eka^* and Lck-Cre *XBP1^lox/lox^/Slfn2^eka/eka^* T cell response to homeostatic proliferation signals. For this end, we labeled wild-type, *Slfn2^eka/eka^*, *CHOP^−/−^/Slfn2^eka/eka^* and Lck-Cre *XBP1^lox/lox^*/*Slfn2^eka/eka^* (CD45.2) splenocytes with the cytosolic dye Cell Trace and adoptively transferred them into sublethally irradiated wild-type (CD45.1) recipients. As expected wild-type T cells underwent proliferation and we detected no *elektra* T cells in the spleen of recipient mice 7 d after infusion (Figure 5C, left panels and Supplementary Figure 3). T cells from both *CHOP^−/−^/Slfn2^eka/eka^* and *Lck-Cre XBP1^lox/lox^*/*Slfn2^eka/eka^* failed to proliferate, however were still detectable in a pretty high percentages (Figure 5C, right panels and Supplementary Figure 3). These results demonstrate that *in vivo*, under lymphopenic environment, *elektra* T cell death is mediated by the XBP1/CHOP pathway. However, the proliferation defect of *elektra* T cells cannot be attributed only to these proteins.

## DISCUSSION

The outcome of an immune response is dependent on the balance between stimulatory and inhibitory signals. This delicate balance is critical for the ability of the immune system to defend the host, while maintaining tolerance to self so to prevent autoimmunity and immunopathology. To achieve this, the immune system maintains a vast repertoire of immunologically naïve lymphocytes in a quiescent state characterized by inactive cell cycle (arrest in G0), small cell size and relatively low basal metabolic activity [17]. Intuitively, it may be expected that elimination of a regulator of quiescence would have led to hyperactive immune responses and even autoimmunity. However, several recent findings demonstrated that the exact opposite can also happen; disruption of quiescence leads to immunodeficiency due to loss of proliferation capabilities and susceptibility to cell death of T cells [23, 31–34]. These results suggest that quiescence programming has a broader role in immunity than previously recognized, and that quiescence rather than a passive process is an active process that enforces passiveness. Nevertheless, most of the factors and pathways that maintain immune cell quiescence have yet to be identified.

Naïve quiescent T cells favor energy production over biosynthesis. To accommodate for this need, naïve T cells rely predominantly on the high-energy-yielding mitochondrial metabolism in which metabolites are oxidized via the TCA cycle [18, 35]. Upon activation, T cells undergo dramatic shift in cell metabolism switching from oxidative metabolism to aerobic glycolysis, to support their expansion and effector functions [18, 35].

The Forkhead Box O1 (FOXO1) [31, 33] and tuberous sclerosis 1 (Tsc1) [32, 34] are both well-established quiescence maintaining factors in T cells. Both proteins play a major role in controlling the metabolic shift that is needed for the transition from quiescence to activation. In addition to their role in metabolism, FOXO1 and Tsc1 have a major role in protecting cells from oxidative stress [36] or ER stress [37] respectively. These findings strongly imply for an intimate relationship between quiescence, metabolism and stress signaling. Therefore it plausible that loss of quiescence will be accompanied by development of stress conditions to be met by compensatory responses. This balance can have a substantial influence on cell fate decisions.

Here we showed that *elektra* T cells display chronic ER stress under steady state conditions (Figure 2). Modulation of the ER stress response by depletion of either XBP1 or CHOP restored the viability (Figure 3) and partially improved the developmental abnormalities and proliferation capabilities of *elektra* T cells (Figures 4 and 5). These results establish a functional connection between the loss of quiescence in *elektra* T cells and chronic unresolved ER stress.

Previous attempts to rescue *elektra* T cell viability either by over-expression of BCL2 [23] or p53 deficiency [24] demonstrated that although the propensity to undergo apoptosis was reduced, *elektra* T cells displayed a semiactivated phenotype and were unable to proliferate. These results strongly indicate that the *elektra* T cell death is a consequence of loss of quiescence. The results presented here demonstrate that knockout of CHOP or XBP1 not only prevented *elektra* T cell death, but also partially prevented the semiactivated phenotype and partially restored the T cell proliferative capacity upon activation (Figures 3 and 5).

Deletion of the tuberous sclerosis complex 1 (Tsc1) gene leads to a substantial reduction in peripheral T cell numbers [32, 37], which correlates with increased propensity to apoptosis that can be rescued by expression of Bcl-2 [37]. Moreover, Tsc1 deficiency in T cells leads to acquisition of a semiactivated phenotype caused by loss of quiescence [37]. This phenotype highly resembles the phenotype observed in *elektra* T cells.

Tsc1, together with its partner Tsc2, restrains the activation of mammalian target of rapamycin (mTOR), an important regulator of metabolism and translation that is typically induced following PI3K signaling [38]. In the context of tumors and liver cells, ER stress and activation of the UPR pathway are important pathological features of TSC deficiency; TSC1 and TSC2 deficiency induces ER stress and activates the UPR [37]. The resulting ER stress increases the vulnerability of various cell lines and tumors to apoptosis [37]. Although not validated in TSC1 KO T cells, ER stress may also contribute to the quiescence defect in this model.

The functional connection between the loss of quiescence in Slfn2-deficiency and chronic unresolved ER stress is not known. As noted above, the *elektra* phenotype is caused by a point mutation in Slfn2 gene. Therefore, one may argue that the *elektra* mutation possibly results in misfolding and aggregation of the mutated Slfn2 protein leading to the observed ER stress. However, *elektra* heterozygous mice do not demonstrate any of the defects observed in *elektra* homozygous mice [23]. In addition, other cells than T cells and monocytes, which also express Slfn2 (e.g. granulocytes, B cells and NK cells), are not influenced by the *elektra* mutation [23]. Furthermore, Slfn2 overexpression in *elektra* homozygous completely rescues the entire phenotypes of the *elektra* mice [23]. Finally, knockdown of Slfn2 by shRNA recapitulate the *elektra* phenotype in EL4 cells [24]. These evidences strongly suggest that the ER stress, as well as other aspects of the *elektra* phenotype, is caused by the loss-of-function of the Slfn2 rather than by a gain-of-function caused by misfolding of the protein. Therefore, we propose two options for the underlying mechanism; one possibility suggests that Slfn2 directly regulates ER homeostasis. According to this possibility the UPR should be a main defect in *elektra* cells. In this regard, Slfn2 can play a role in protein folding in the ER (chaperone), protein translocation into the ER and protein trafficking within and downstream to the ER. A second possibility is that the observed chronic ER stress in *elektra* cells is a consequence of an overall much broader phenotype caused by Slfn2 deficiency. For example; an unbalanced translational regulation [39], impaired lipid and cholesterol biosynthesis and trafficking [40], disturbances of redox homeostasis [41], glucose deprivation [42] and aberrant Ca^+2^ regulation [43]. All of these conditions also result in ER stress; however, UPR is only a part of the effect. Interestingly, the mechanism by which TSC deficiency results in ER stress induced cell death was attributed to increased mTOR activity [37], which triggers uncontrolled protein synthesis. Moreover, Li, M. *et al*. demonstrated that the human SLFN family member, Slfn11, inhibits retroviral protein translation in a codon-usage dependent manner [44]. Considering these results and our findings, it is appealing to suggest that in *elektra* cells the chronic ER stress is mediated due to loss of protein translational control which leads among several other defects also to inability to match protein load to the folding capacity of the ER, supporting the second option. This scenario suggests a potential regulatory role for Slfn2 in protein translation. Therefore, models that directly explore the role of protein translation in T cell quiescence should be developed.

## MATERIALS AND METHODS

### Mice

*Slfn2^eka/eka^* mice were previously generated as described in Berger *et al*. [23]. The C57BL/6J (wild-type), CHOP^−/−^, C57BL/6.SJL (PtprcaPep3b; Ly5.1) (CD45.1) and B6.Cg-Tg(Lck-icre)3779Nik/J (Lck-Cre) mice were from The Jackson Laboratory. The T cell specific *XBP1* KO mice were generated by crossing mice containing a conditional floxed allele of XBP-1 (*XBP-1^lox/lox^*, described in [45]) with transgenic mice expressing Cre under the control of the Lck gene promoter (Lck-Cre). Mice were maintained and bred under specific pathogen free conditions in the Hebrew University animal facilities according to Institutional Animal Care and Use Committee regulations. All mice were maintained on the C57BL/6 background and used for experiments at 8–12 weeks of age.

### Real time PCR

Total RNA from purified monocytes or T cells was extracted with Direct-zol RNA MiniPrep Plus (Zymo Research). cDNA was synthesized using ProtoScript First Strand cDNA Synthesis Kit (Neb). Quantitative real-time PCR was then performed using QuantStudio 12K Flex Real Time PCR system with a Power SYBR green PCR master mix kit (Applied Biosystems). The primers sequences used in this study are previously described [46].

### Flow cytometry

Spleen cells were stained with various conjugated mAbs against cell-surface markers. In some of the experiments, following the cell surface staining, cells were fixed, permeabilized and stained with intracellular antibody using Leucoperm-kit (AbD Serotec) according to manufacturer instructions. ER tracker staining was performed by incubating cells with 1μM ER-Tracker blue-white DPX (Molecular Probes) diluted in Hank's Balanced Salt Solution for 30 minutes in 37^°^C. Stained cells were analyzed by Gallios flow cytometer with Kaluza software (Beckman Coulter).

### Inflammatory monocytes and T cells isolation

For monocytes isolation total bone marrow cells were harvested and inflammatory monocytes were isolated by EasySep mouse monocyte isolation kit (Stem Cell Technologies). For further purification, CD11b^+^ Ly6C^high^ monocytes were sorted by flow cytometry. Total T cells were isolated from spleen by EasySep mouse T cell isolation kit (Stem Cell Technologies).

### Adoptive transfer of T cells

A total of 1×10^7^ CD45.2 splenocytes were labeled with Cell-trace violet (Molecular Probes c34557) and IV injected into CD45.1 C57BL/6J recipient mice that had been sublethally irradiated (400 rads) 24 h earlier. 7 days after adoptive transfer, spleen cells were harvested, stained for CD45.2, CD8 and CD4 and analyzed by flow-cytometry for cell-trace dilution.

### T cell proliferation assay

A total of 2.5 × 10^6^ cell-trace violet-labeled spleen cells were activated in 24-flat-well plates previously bounded with anti-CD3ε (2μg/ml) and anti-CD28 (1μg/ml) and IL-2 (20ng/ml). The cells were analyzed 72 h after activation by flow cytometry as described in the adoptive transfer section.

### Antibodies

The following antibodies were used for flow cytometry: anti-CD8α (53-6.7), anti-CD4 (L3T4), anti-CD45.1 (A20), anti-CD45.2 (104), anti-Bcl-2 (10C4), anti-CD44 (IM7), CD122 (IL2RB) (5H4), CD127 (IL7R) (SB/199), anti-CD11b (M1/70), and anti-Ly6C (AL-21; Biolegend). Purified anti-CD3ε (145–2C11) and anti-CD28 (37.51; both from Biolegend) were used at the appropriate concentration for T cell activation.

## SUPPLEMENTARY MATERIAL FIGURES


