# Supplementary material for: Schlafen2 mutation unravels a role for chronic ER stress in the loss of T cell quiescence
Source: Oncotarget. 2016 Jun 3;7(26):39396–407. doi: 10.18632/oncotarget.9818 (PMC5129940; doi:10.18632/oncotarget.9818)
Supplement: Supplementary file 1 [file oncotarget-07-39396-s001.pdf]

Schlafen2 mutation unravels a role for chronic ER stress in the loss of T cell quiescence

Supplementary Material

This PDF file includes:

Figures. S1 to S3

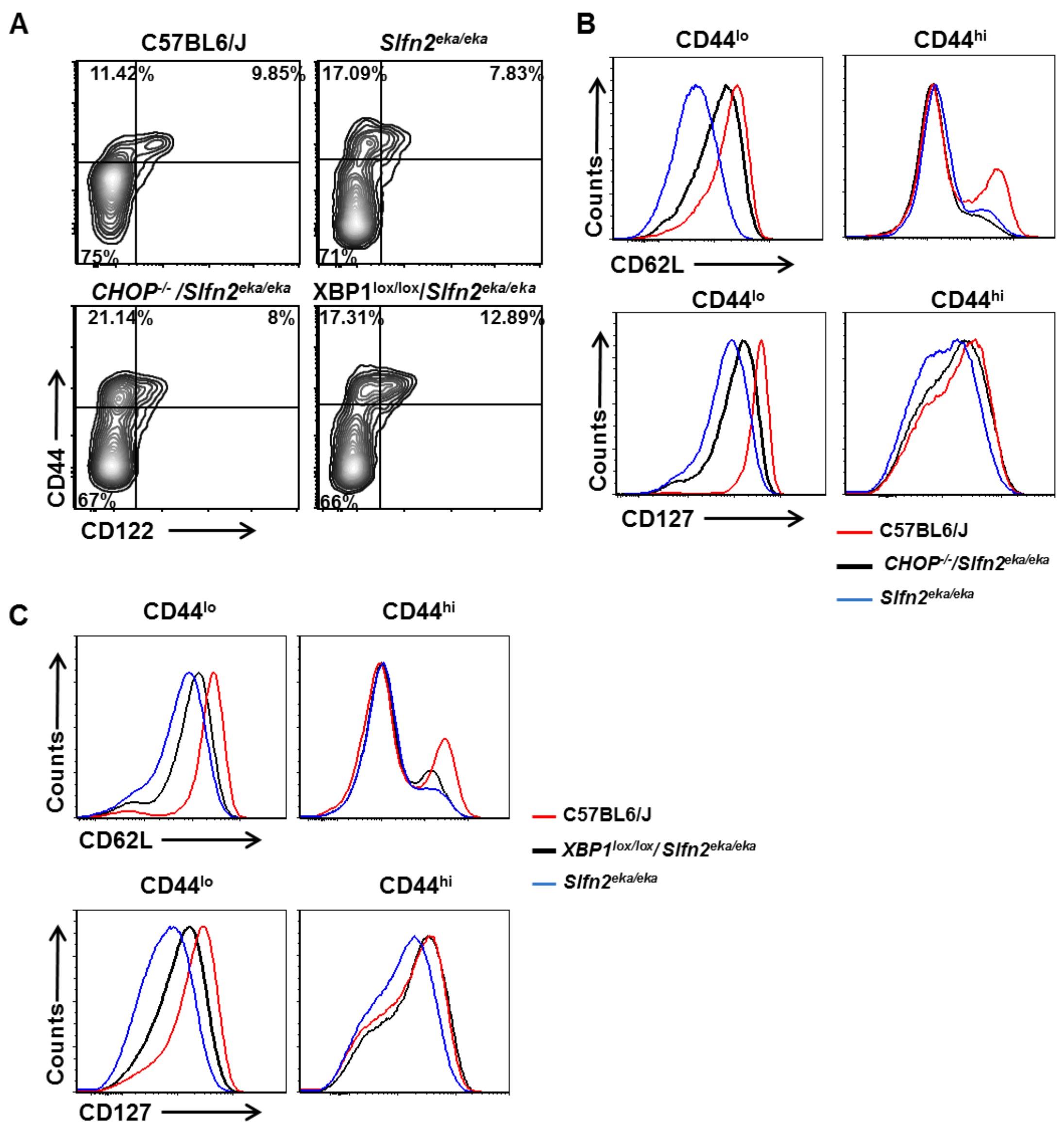

Figure S1: CHOP or XBP1 deficiency partially rescues the (semi-activation) phenotype of Slfn2$^{eka/eka}$ CD4^{+} T cells. (A) Flow cytometry analysis of the staining for CD122 (IL-2Rβ) and CD44 in splenic CD4^{+} T cells from C57BL6/J, *Slfn2*$^{eka/eka}$, *CHOP*$^{-/-}$*/Slfn2*$^{eka/eka}$ or XBP1lox/lox/Slfn2eka/eka mice (n = 9). Numbers in quadrants indicate percent cells in each. (B,C) Flow cytometry analysis for the expression levels of CD62L (upper panel) and IL-7Rα (CD127) (lower panel) on surface of both CD4^{+}CD44^{lo} (left) and CD4^{+}CD44^{hi} (right) T cells from the C57BL6/J, *Slfn2*$^{eka/eka}$, *CHOP*$^{-/-}$*/Slfn2*$^{eka/eka}$ (B) or *XBP1*$^{lox/lox}$*/Slfn2*$^{eka/eka}$ mice (C) (n=9).

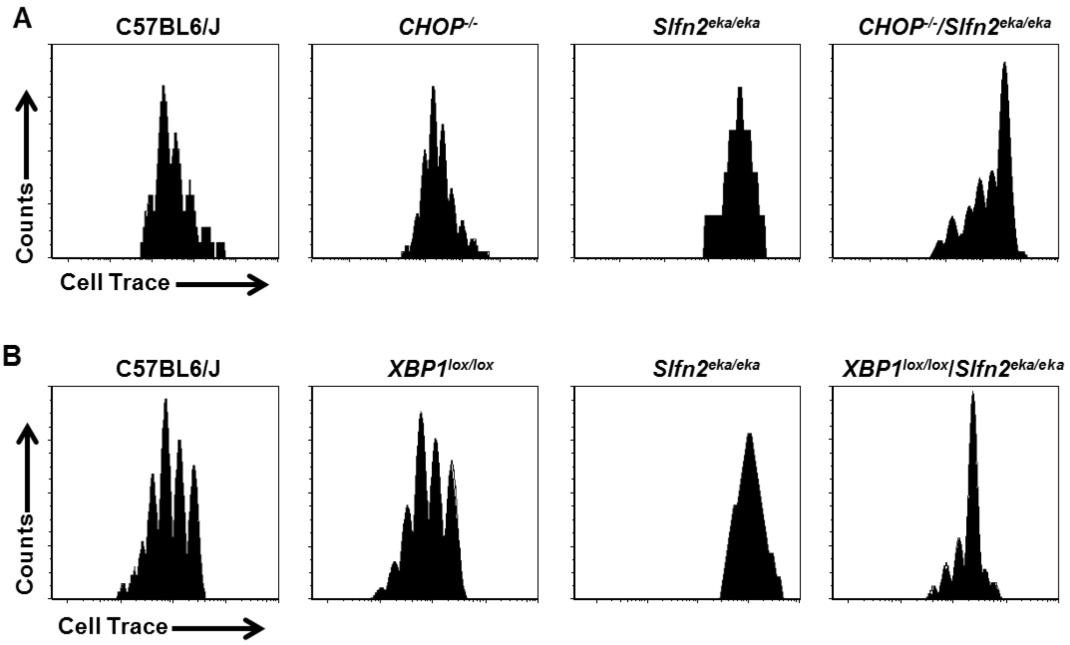

Figure S2: CHOP or XBP1 deficiency partially rescues *Slfn2*$^{eka/eka}$ CD4^{+} T cells proliferation capacity upon activation ex vivo. (A, B) Cell trace dilution of splenic CD4^{+} obtained from C57BL6/J, *Slfn2*$^{eka/eka}$, *CHOP*$^{-/-}$/*Slfn2*$^{eka/eka}$ (A) or *XBP1*$^{lox/lox}$/*Slfn2*$^{eka/eka}$ (B) mice stimulated for 72 hours with plate bound anti-CD3ε (2µg/ml) plus anti-CD28 (1µg/ml) and IL-2 (20ng/ml) (n=6 each genotype). Histograms are gated on CD4^{+}.

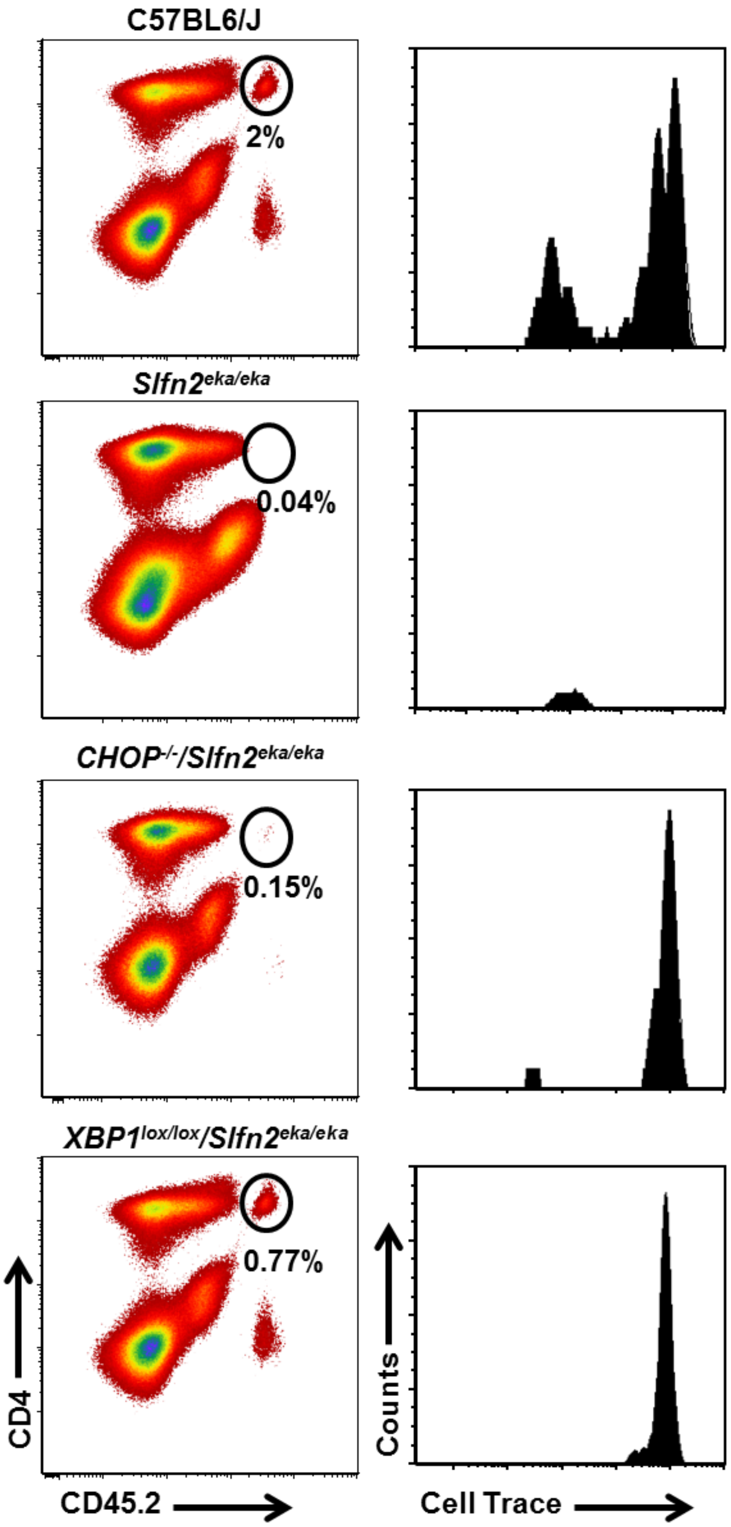

Figure S3: CHOP and XBP1 deficiency partially rescue *Slfn2*$^{eka/eka}$ CD4^{+} T cells death in lymphopenic environment in vivo. Cell trace dilution of splenic CD4^{+} obtained from CD45.2 C57BL6/J, *Slfn2*$^{eka/eka}$, CHOP$^{-/-}$/Slfn2$^{eka/eka}$ or *XBP1*$^{lox/lox}$*/Slfn2*$^{eka/eka}$ adoptively transferred into irradiated (400 rad) CD45.1 C57BL6/J mice for 7 days (n=5). Numbers on plots indicate percentages of donor survived CD4^{+} T cells. Histograms are gated on CD45.2^{+} CD4^{+}.
